# Microtubule Stabilization by Mdp3 Is Partially Attributed to Its Modulation of HDAC6 in Addition to Its Association with Tubulin and Microtubules

**DOI:** 10.1371/journal.pone.0090932

**Published:** 2014-03-10

**Authors:** Tala, Xiaoou Sun, Jie Chen, Linlin Zhang, Ningning Liu, Jun Zhou, Dengwen Li, Min Liu

**Affiliations:** 1 Department of Genetics and Cell Biology, College of Life Sciences, Nankai University, Tianjin, China; 2 Department of Biochemistry, School of Basic Medical Sciences, Tianjin Medical University, Tianjin, China; Baylor College of Medicine, United States of America

## Abstract

Microtubule-mediated cellular events such as intracellular transport and the maintenance of cell polarity are highly dependent upon microtubule stability, which is controlled by a repertoire of microtubule-associated proteins (MAPs) in the cell. MAP7 domain-containing protein 3 (Mdp3) has recently been identified as a critical regulator of microtubule stability. However, it remains elusive how Mdp3 carries out this function. In this study, by examination of tubulin partitioning between the polymer and soluble dimer forms, we found that Mdp3 could protect microtubules from cold- or nocodazole-induced depolymerization. Immunoblotting and immunofluorescence microscopy showed that knockdown of Mdp3 expression significantly reduced the level of tubulin acetylation. In vitro tubulin polymerization assays revealed that the amino-terminal region of Mdp3 was necessary for its ability to stabilize microtubules. Immunoprecipitation and pulldown experiments showed that the amino-terminal region mediated the interaction of Mdp3 with histone deacetylase 6 (HDAC6), in addition to its association with tubulin and microtubules. Immunofluorescence microscopy further demonstrated that endogenous Mdp3 and HDAC6 colocalized in the cytoplasm. Moreover, depletion of Mdp3 dramatically increased the activity of HDAC6 toward tubulin deacetylation. These findings suggest that Mdp3 controls microtubule stability through its binding to tubulin and microtubules as well as its regulation of HDAC6 activity.

## Introduction

Microtubules are one of the major cytoskeletal components and play critical roles in diverse cellular events. While the dynamic property of microtubules is important for many microtubule-mediated cellular events such as cell division and migration [1], [2], the stability of microtubules is also important, especially for intracellular transport and the maintenance of cell polarity [3], [4]. Deregulation of microtubule stability can lead to severe consequences such as developmental defects and neurodegenerative diseases [5]. In cells, microtubule stability is controlled by a number of proteins interacting with microtubules and/or its tubulin subunits, collectively known as microtubule-associated proteins (MAPs) [6], [7]. Notable proteins of the MAP family include MAP1, MAP2, MAP4, tau, and MAP7. While MAP4 is ubiquitously expressed in many cell types, MAP1, MAP2, and tau are largely confined to nerve cells and MAP7 is predominantly expressed in epithelial cells [6], [8].

MAP7 domain-containing protein 3 (Mdp3), also known as MAP7D3, was initially identified in a mass spectrometry-based proteomic analysis as a mitotic spindle component [9], and has recently been demonstrated to interact with both tubulin and microtubules [10]. The Mdp3 gene is located at Xq26.3 and encodes a protein consisting of 876 amino acids. The level of Mdp3 varies significantly in different tissues, with relatively high expression in skeletal muscle and lung tissues [10]. In addition, Mdp3 expression undergoes dramatic changes during the cell cycle, with lower expression in the G2 phase compared with its expression in the other phases of the cell cycle [10]. It has been demonstrated previously that Mdp3 plays an important role in the regulation of microtubule stability [10]. However, the molecular mechanisms underlying this function of Mdp3 remain unclear. In this study, we provide the first evidence that Mdp3 modulates microtubule stability via two different mechanisms, direct binding to microtubules/tubulin and regulating histone deacetylase 6 (HDAC6)-mediated tubulin deacetylation.

## Materials and Methods

### Chemicals and antibodies

Nocodazole, 4′,6-diamidino-2-phenylindole (DAPI), and antibodies against acetylated α-tubulin, glutathione S-transferase (GST), and maltose-binding protein (MBP) were purchased from Sigma-Aldrich. Antibodies against α-tubulin and HDAC6 were from Abcam, and the antibody against GFP were from Roche. The anti-Mdp3 antibody was generated as described previously [10]. Horseradish peroxidase-conjugated secondary antibodies were purchased from Santa Cruz Biotechnology, and fluorescein- and rhodamine-conjugated secondary antibodies were from Jackson ImmunoResearch Laboratories.

### Plasmids, proteins, and siRNAs

Mammalian expression plasmids for GFP-tagged or GST-tagged Mdp3 (full-length and mutants) were generated by using the pEGFPC1 and pEBG vectors, respectively. The mammalian expression plasmid for GFP-HDAC6 was generated using the pEGFPN1 vector. Bacterial expression plasmids for MBP-Mdp3 and various mutants were constructed using the pMALp2T vector. MBP and MBP-Mdp3 fusion proteins were purified with the amylose resin following the manufacturer's protocol (New England Biolabs). MAP-free tubulin and MAP-rich tubulin were purchased from Cytoskeleton. Control (luciferase) and Mdp3 siRNAs were synthesized by Dharmacon.

### Cell culture and transfection

HeLa cells and human umbilical vein endothelial cells (HUVECs) were obtained from the American Type Culture Collection and cultured in DMEM and RPMI 1640 media respectively, supplemented with 2 mM L -glutamine and 10% fetal bovine serum at 37°C in a humidified atmosphere with 5% CO_2_. Plasmids were transfected into cells with the Entranster-D reagent (Engreen Biosystem), and siRNAs were transfected with the DharmaFect1 reagent (Dharmacon), according to the manufacturers' protocols.

### Immunoblotting

Proteins were resolved by SDS-PAGE and transferred onto polyvinylidene difluoride membranes (Millipore). The membranes were blocked in Tris-buffered saline containing 0.2% Tween 20 and 5% fat-free dry milk and incubated first with primary antibodies and then with horseradish peroxidase-conjugated secondary antibodies. Specific proteins were visualized with enhanced chemiluminescence detection reagent according to the manufacturer's instruction (Pierce).

### Immunofluorescence microscopy

Cells grown on glass coverslips were fixed with methanol for 5 min at −20°C or fixed with 4% paraformaldehyde for 30 min at room temperature followed by permeabilization in 0.5% Triton X-100/phosphate-buffered saline (PBS) for 20 min. Cells were then blocked with 2% bovine serum albumin in PBS and incubated in succession with primary antibodies and fluorescein- or rhodamine-conjugated secondary antibodies followed by staining with DAPI for 5 min. Coverslips were mounted with 90% glycerol in PBS and examined with an Axio Observer A1 fluorescence microscope (Carl Zeiss).

### Immunoprecipitation and pulldown

Cell lysates or protein mixtures were incubated with antibody-, glutathione-, or amylase-conjugated agaorse beads at 4°C for 2 h. The beads were washed extensively and boiled in SDS loading buffer, and the precipitated proteins were detected by SDS-PAGE and immunoblotting.

### Examination of tubulin partitioning

Cells were placed on ice or treated with 100 ng/ml nocodazole. Cellular soluble proteins were then extracted with the PEMG buffer (100 mM PIPES, 1 mM EGTA, 1 mM MgSO_4_, 1 mM GTP, pH 6.8) containing 0.1% Triton X-100 and 4 M glycerol. The remaining polymeric (cytoskeletal) fraction was dissolved in 0.5% SDS and 25 mM Tris (pH 6.8). The soluble and polymeric fractions were then subjected to SDS-PAGE and examined by immunoblotting with the anti-α-tubulin antibody.

### *In vitro* tubulin polymerization assay

Rhodamine-labeled tubulin was incubated with purified MBP or MBP-Mdp3 fusion proteins at 37°C for 20 min, and the intensity and length of microtubules were examined with the fluorescence microscope. To examine microtubule stability in vitro, microtubules formed above were placed on ice, and the morphology of microtubules was then examined with the fluorescence microscope.

### *In vitro* tubulin deacetylation assay

Cells were transfected with GFP-HDAC6, and immunoprecipitation was performed with the anti-GFP antibody. The GFP-HDAC6 immunoprecipitate was then incubated with microtubules preformed with MAP-rich tubulin. The levels of acetylated α-tubulin, α-tubulin, and GFP-HDAC6 in the above mixture were then examined by immunoblotting with antibodies against acetylated α-tubulin, α-tubulin, and GFP, respectively.

### Statistics

Results are expressed as the mean ± S.E. Statistical analyses were performed using the Student's t-test.

## Results

**Figure 1:**
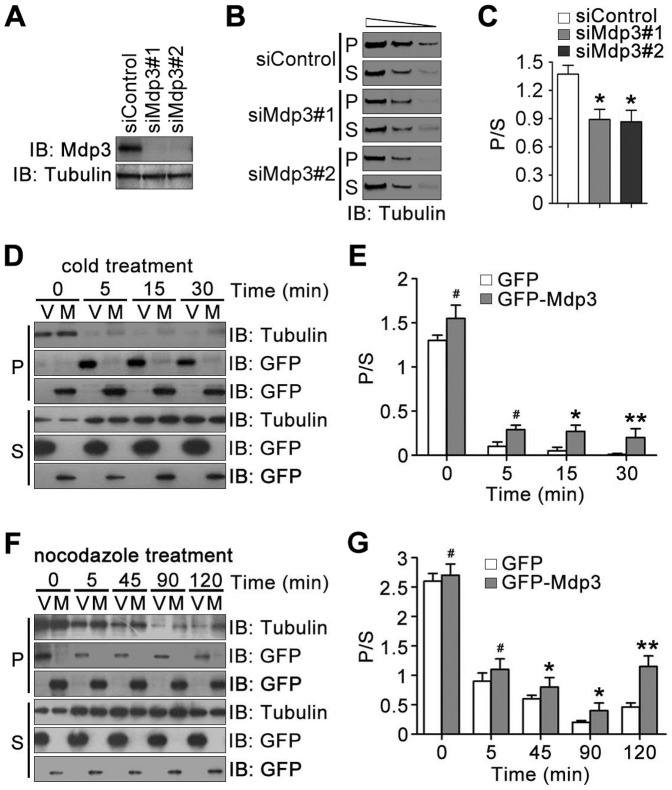
Mdp3 protects microtubules from cold- or nocodazole-induced depolymerization. (A) HeLa cells were transfected with control or Mdp3 siRNAs. The levels of Mdp3 and α-tubulin were then examined by immunoblotting with antibodies against Mdp3 and α-tubulin. (B) HeLa cells were transfected with control or Mdp3 siRNAs, placed on ice for 30 min, and then re-incubated at 37°C for 30 min to permit microtubule regrowth. Tubulin partitioning between the polymer (P) and soluble dimer (S) forms was examined by immunoblotting with the anti-α-tubulin antibody. Each of the polymeric and soluble fractions was loaded at a 3-fold serial dilution to ensure the intensity of bands on the blots in the linear range of detection. (C) Experiments were performed as in (B), and the ratio of tubulin polymer to soluble dimer was analyzed. (D) HeLa cells were transfected with GFP (V) or GFP-Mdp3 (M) and placed on ice for 0, 5, 15, or 30 min. Tubulin partitioning between the polymer and soluble dimer forms was examined by immunoblotting with the anti-α-tubulin antibody, and the expression of GFP and GFP-Mdp3 was examined by immunoblotting with the anti-GFP antibody. (E) Experiments were performed as in (D), and the ratio of tubulin polymer to soluble dimer was analyzed. (F) HeLa cells were transfected with GFP or GFP-Mdp3 and treated with nocodazole for 0, 5, 45, 90, or 120 min. Tubulin partitioning between the polymer and soluble dimer forms was examined by immunoblotting with the anti-α-tubulin antibody, and the expression of GFP and GFP-Mdp3 was examined by immunoblotting with the anti-GFP antibody. (G) Experiments were performed as in (F), and the ratio of tubulin polymer to soluble dimer was analyzed. *, *p*<0.05; **, *p*<0.01; #, not significant (*p*≥0.05).

To verify the function of Mdp3 in stabilizing microtubules, we performed cold recovery assay using HeLa cells and then examined by immunoblotting the partitioning of cellular tubulin between the polymer and soluble dimer forms. Immunoblotting revealed that Mdp3-specific siRNAs decreased its expression effectively (Fig. 1A). Mdp3 siRNAs significantly reduced the ratio of tubulin polymer to soluble dimer (Fig. 1B and 1C), indicating a role for Mdp3 in promoting the stability of cellular microtubules. To corroborate the above finding, we overexpressed Mdp3 in HeLa cells and then performed immunoblotting to analyze tubulin partitioning upon cold treatment. Microtubules depolymerized dramatically after cells were placed on ice, and Mdp3 modestly, but significantly, shifted tubulin partitioning toward the polymer form (Fig. 1D and 1E). In addition, the majority of Mdp3 was detected to associate with the polymer form of tubulin (Fig. 1D). We also investigated the effect of Mdp3 overexpression on tubulin partitioning after cells were treated with the microtubule-depolymerizing agent nocodazole. Consistent with the cold treatment results, Mdp3 could protect microtubules from nocodazole-induced depolymerization (Fig. 1F and 1G). Mdp3 also predominantly resided with the polymer form of tubulin (Fig. 1F).

**Figure 2:**
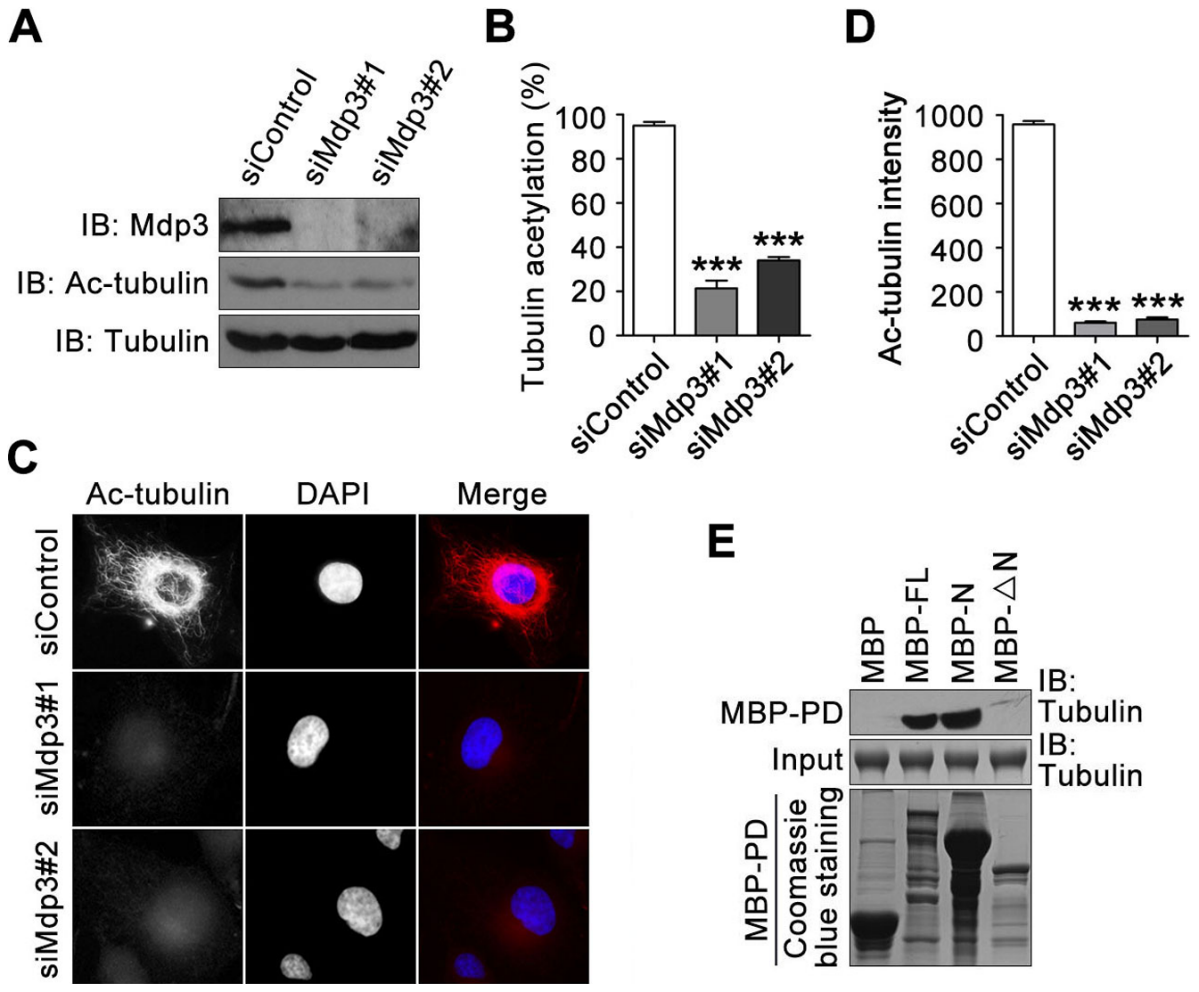
Knockdown of Mdp3 expression reduces the level of tubulin acetylation. (A) HeLa cells were transfected with control or Mdp3 siRNAs. The levels of Mdp3, acetylated α-tubulin, and α-tubulin were then examined by immunoblotting with antibodies against Mdp3, acetylated α-tubulin, and α-tubulin. (B) Experiments were performed as in (A), and the level of tubulin acetylation was determined by dividing the density of acetylated α-tubulin by the density of α-tubulin. The data were then normalized to the control group. (C) HUVECs were transfected with control or Mdp3 siRNAs, stained with anti-acetylated α-tubulin antibody (red) and then the DNA dye DAPI (blue), and visualized under the fluorescence microscope. (D) Experiments were performed as in (C), and the level of tubulin acetylation was analyzed by measuring the fluorescence intensity of acetylated α-tubulin. (E) MAP-free tubulin was incubated with bacterially purified MBP or MBP-Mdp3 fusion proteins immobilized on amylase-conjugated agarose beads. The levels of tubulin in the MBP-pulldown (PD) preparation and in the input were examined by immunoblotting with the anti-α-tubulin antibody. The levels of MBP and MBP-Mdp3 fusion proteins used in the assays were examined by Coomassie blue staining. FL, full-length; N, amino-terminal region; ΔN, without the amino-terminal region. ***, *p*<0.001.

To gain mechanistic insight into the promotion of microtubule stability by Mdp3, we examined the effect of Mdp3 siRNAs on the level of tubulin acetylation in HeLa cells. Using an antibody specifically for acetylation α-tubulin, we found that siRNA-mediated knockdown of Mdp3 expression resulted in a significant reduction of tubulin acetylation level (Fig. 2A and 2B).

We then performed immunofluorescence microscopy in HUVECs to further investigate the effect of Mdp3 on tubulin acetylation. As shown in Fig. 2C and 2D, Mdp3 siRNAs led to a remarkable decrease in the fluorescence intensity of acetylated tubulin. The immunoblotting and immunofluorescence microscopy experiments together indicate that Mdp3 could enhance tubulin acetylation.

To have a better understanding of Mdp3 activity toward the stability of microtubules, we purified from bacteria MBP-tagged Mdp3 full-length (FL) and two mutant forms, one containing the amino-terminal region only (N) and the other lacking the amino-terminal region (ΔN). These proteins were immobilized on amylase-conjugated agarose beads and then incubated with MAP-free tubulin. By MBP-pulldown and immunoblotting assays, we found that tubulin was present in the MBP-pulldown preparations of Mdp3-FL and Mdp3-N, but not Mdp3-ΔN (Fig. 2E), suggesting the requirement for the amino-terminal region for its binding to tubulin.

**Figure 3:**
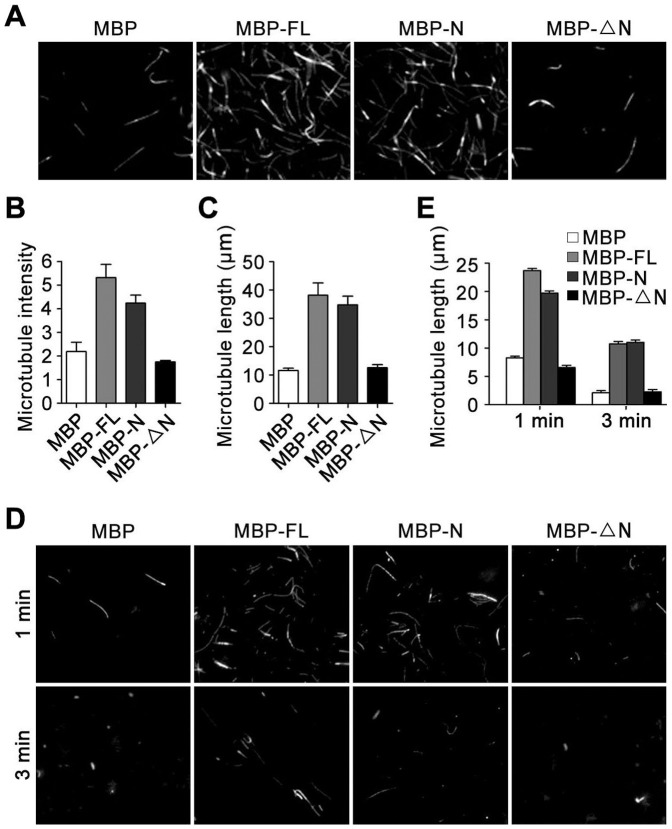
The amino-terminal region is necessary for Mdp3 to stabilize microtubules. (A) Rhodamine-labeled tubulin was incubated with purified MBP or MBP-Mdp3 fusion proteins at 37°C for 20 min, and the morphology of microtubules was examined with the fluorescence microscope. (B and C) Experiments were performed as in (A), and the intensity (B) and length (C) of microtubules were measured. (D) Rhodamine-labeled tubulin was incubated with purified MBP or MBP-Mdp3 fusion proteins at 37°C for 20 min and then placed on ice for 1 or 3 min, and the morphology of microtubules was examined with the fluorescence microscope. (E) Experiments were performed as in (D), and the length of microtubules was measured.

To further investigate the role of the amino-terminal region in mediating the effect of Mdp3 on microtubule stability, we performed in vitro tubulin polymerization assays. We incubated MBP or MBP-Mdp3 fusion proteins with rhodamine-labeled tubulin at 37°C to allow the assembly of tubulin into microtubules. By fluorescence microscopic examination of the microtubules formed in the above assays, we found that Mdp3 dramatically increased the intensity of microtubules and that the N form of Mdp3 had a modest effect; in contrast, this effect of Mdp3 was completely abrogated in the ΔN form (Fig. 3A and 3B). Further analyses revealed that Mdp3-FL and Mdp3-N, but not Mdp3-ΔN, remarkably increased the length of microtubules (Fig. 3A and 3C).

We also placed microtubules assembled in the above purified system on ice to allow microtubule disassembly and then examined the morphology of microtubules with the fluorescence microscope. As shown in Fig. 3D and 3E, Mdp3-FL and Mdp3-N, but not Mdp3-ΔN, could protect microtubules from cold-induced depolymerization. Collectively, the above findings demonstrate that the amino-terminal region of Mdp3 mediates its activity to stabilize microtubules.

**Figure 4:**
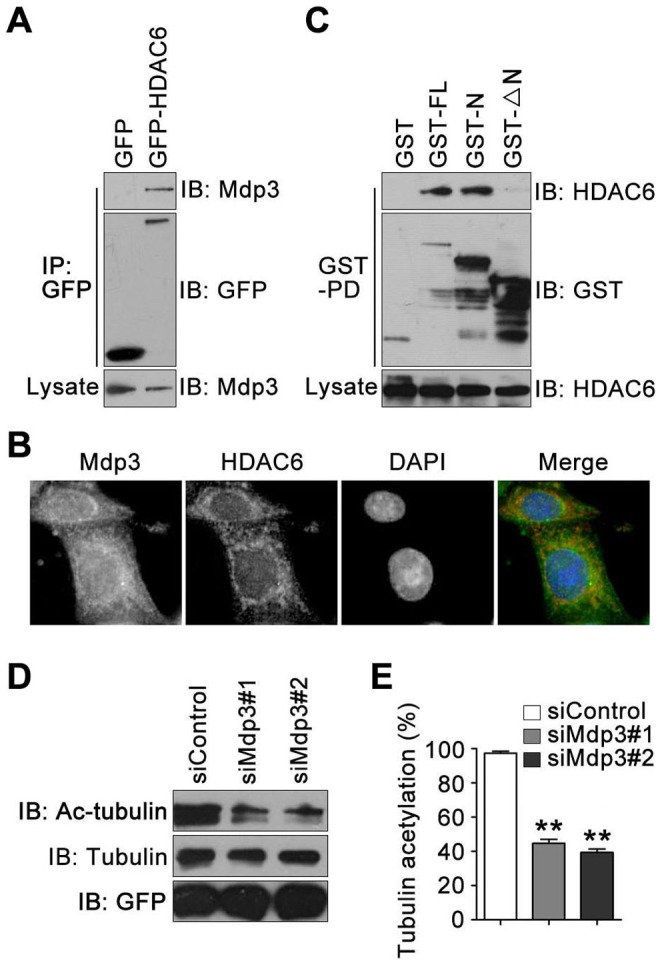
Mdp3 interacts with HDAC6 and inhibits HDAC6 activity. (A) HeLa cells were transfected with GFP or GFP-HDAC6, and anti-GFP immunoprecipitation was performed. The immunoprecipitates and cell lysates were then immunoblotted with anti-Mdp3 or anti-GFP antibodies. (B) HUVECs were stained with anti-Mdp3 (green) and anti-HDAC6 (red) antibodies and the DNA dye DAPI, and images were then taken under the fluorescence microscope. (C) HeLa cells were transfected with GST or GST-Mdp3 fusion proteins, and GST-pulldown assay was performed. The pulldown preparation and cell lysates were then immunoblotted with anti-HDAC6 or anti-GST antibodies. (D) HeLa cells were transfected with GFP-HDAC6 and control or Mdp3 siRNAs, and anti-GFP immunoprecipitation was performed. The GFP-HDAC6 immunoprecipitate was then incubated with preformed microtubules. The levels of acetylated α-tubulin, α-tubulin, and GFP-HDAC6 in the above mixture were then examined by immunoblotting with antibodies against acetylated α-tubulin, α-tubulin, and GFP, respectively. (E) Experiments were performed as in (D), and the level of tubulin acetylation was determined by dividing the density of acetylated α-tubulin by the density of α-tubulin. The data were then normalized to the control group. **, *p*<0.01.

To understand how Mdp3 enhances tubulin acetylation, we studied whether Mdp3 modulates the activity of the tubulin acetylase HDAC6 [11]–[13]. We first examined the potential interaction of these two proteins. By immunoprecipitation and immunoblotting assays, we found that Mdp3 was present in the GFP-HDAC6 immunoprecipitates in HeLa cells (Fig. 4A). In addition, by immunofluorescence microscopy, we observed the colocalization between endogenous Mdp3 and HDAC6 in HUVECs (Fig. 4B). GST-pulldown and immunoblotting assays further revealed that HDAC6 existed in the pullown preparations of Mdp3-FL and Mdp3-N, but not Mdp3-ΔN (Fig. 4C).

We then sought to analyze whether Mdp3 affected the tubulin deacetylase activity of HDAC6. To test this, we transfected HeLa cells with GFP-HDAC6 and control or Mdp3 siRNAs, and then incubated the GFP-HDAC6 immunoprecipitate with preformed microtubules. By this in vitro tubulin deacetylation assay, we found that knockdown of Mdp3 expression significantly increased the activity of HDAC6 to deacetylate tubulin (Fig. 4D and 4E). Taken together, these results demonstrate that Mdp3 interacts with HDAC6 and inhibits HDAC6 activity.

## Discussion

Proteins of the MAP family are key players in the regulation of microtubule stability [6], [7]. Abnormal expression or posttranslational modification of MAPs has been implicated in the pathogenesis of human diseases especially neurodegenerative diseases. For example, the phosphorylated form of tau is known to associate with tubulin to stabilize axonal microtubules and promote tubulin polymerization into microtubules, and abnormal phosphorylation of tau results in the destabilization of axonal microtubules, leading to the development of Alzheimer's disease and many other neurodegenerative diseases collectively called tauopathies [5]. Therefore, the elucidation of how various MAPs exert their effects on microtubules may have a profound impact on the diagnosis and therapy of many human diseases.

Mdp3 has recently been characterized as a novel member of the MAP family [10]. Intriguingly, Mdp3 interacts with microtubules/tubulin and stabilizes microtubules through its amino-terminal region instead of its carboxyl-terminal MAP7 domain, indicating the structural diversity of the MAP7 domains present in different proteins. It would be interesting to investigate in the future whether the two closely related proteins, Mdp1 and Mdp2, interact with microtubules/tubulin and promote microtubule stability, and if so, whether these actions are mediated by their MAP7 domains. Further studies are also warranted to study the expression patterns of Mdp3 in different cell types and to analyze whether the expression or posttranslational modification of Mdp3 is altered in various human diseases.

Stable microtubules are known to possess a high level of tubulin acetylation, although the causal relationship between tubulin acetylation and microtubule stability remains to be investigated [14]. The acetyltransferases Elongator and MEC-17 have been reported to catalyze the tubulin acetylation process [15], [16], and conversely the deacetylases HDAC6 and sirtuin 2 (SIRT2) are able to remove the acetyl group from tubulin [11]–[13], [17]. The present study reveals that the amino-terminal region of Mdp3 mediates its interaction with HDAC6 in the cytoplasm, in addition to mediating Mdp3 binding to microtubules/tubulin. Interestingly, Mdp3 suppresses the activity of HDAC6, leading to enhanced tubulin acetylation. This finding suggests that Mdp3 might regulate microtubule stability by acting on HDAC6-mediated tubulin deacetylation, in addition to its direct association with microtubules/tubulin.

HDAC6, as a unique member of the HDAC family with a predominant localization in the cytoplasm, has been implicated in numerous cellular processes such as cell motility, cell-cell interaction, and transcriptional regulation [18], [19]. HDAC6 carries out the above functions primarily through its deacetylation of substrate proteins such as α-tubulin [11]–[13], cortactin [20], and Hsp90 [21]. In addition, HDAC6 could interact with polyubiquitinated misfolded proteins and the dynein motor, facilitating the transport of misfolded proteins to the aggresome [22]. Over the past decade, HDAC6 has emerged as a promising target for drug development due to its involvement in oncogenic cell transformation and neurodegeneration [23]–[26]. In this scenario, our finding that Mdp3 is an endogenous inhibitor of HDAC6 may have important implications in health and diseases.
